# Interpreting Bisphenol A Absorption in the Canine Oral Cavity: Gayrard et al. Respond

**DOI:** 10.1289/ehp.1307424R

**Published:** 2013-12-01

**Authors:** Véronique Gayrard, Marlène Z. Lacroix, Séverine H. Collet, Catherine Viguié, Alain Bousquet-Melou, Pierre-Louis Toutain, Nicole Picard-Hagen

**Affiliations:** INRA (Institut National de la Recherche Agronomique), UMR1331 (Unité Mixe de Recherche 1331), Toxalim (Research Center in Food Toxicology), Université de Toulouse, Toulouse,France, E-mail: pltoutain@wanadoo.fr

The comments by Teeguarden et al. offer us the opportunity to explain some possible pharmacokinetic (PK) and PK/pharmacodynamic (PK/PD) consequences of sublingual bisphenol A (BPA) absorption. Nowhere in our paper (Gayrard et al. 2013) did we report that “nanograms-per-milliliter serum concentrations of BPA resulting from sublingual absorption are plausible” in humans.”

Using elementary PK computation, Teeguarden et al. concluded that peak concentrations in mixed systemic serum should be < 0.1 ng/mL even after a bolus intraveneous (iv) administration based on the 95th percentile of the aggregate daily U.S. human exposure (0.22 μg/​kg body weight). Using the same scaling approach but considering a subpopulation of children 6–11 years of age with a 95th percentile of 0.481 µg/​kg/​day (Lakind and Naiman 2011), and taking into account our own data from dogs, the maximum initial plasma BPA concentration would be about 0.6 ng/mL. More important, what should be highlighted from our study is that, within 1 hr after administration of the low BPA dose (0.05 mg/kg), the BPA concentrations in blood collected from the jugular vein (i.e., downstream from the BPA absorption site) were higher than those measured after the corresponding iv administration (see Figure 1B of Gayrard et al. 2013). Teeguarden et al. should not be confused by the question of validity of a sampling site for making a sound pharmacokinetic computation and the biological relevance of a local concentration that is a biological fact that needs to be considered. Scaling our local jugular concentrations by human BPA exposure (0.22 μg/kg) leads to the computation of a jugular BPA concentration > 1 ng/​mL. What is true for sublingual absorption and the jugular vein could be true for any sampling site located downstream of any other absorption site, such as the human cubital vein after percutaneous absorption of BPA from thermal receipt paper, leading to serum BPA concentrations > 1 ng/mL (vom Saal FS, personal communication). This means that high BPA concentrations that are neither contamination (vom Saal 2013) nor a violation of PK principles can be observed in humans.

More generally, it is our opinion that it is unwise to consider that “mixed systemic serum following sublingual exposure” and blood samples obtained “from a site reflecting systemic exposure” are the only valuable ways to describe the PK of BPA and to discuss the PK/PD relationship. The buccal absorption of BPA can be viewed as a direct infusion of BPA into the internal jugular vein (in humans) likely to lead downstream to a totally different pattern of BPA biophase exposure compared with intestinal absorption. Buccal absorption should generate a series of BPA peaks (associated with meals) and troughs (during the interdigestive period) in the blood rather than a low steady exposure following intestinal absorption. In addition, the amplitude of these intermittent jumps in BPA concentration could be higher in arterial blood that those measured peripherally. Chiou (1989) reported that after iv administration, measured peripheral venous concentrations could be initially lower or much lower than the corresponding arterial concentrations, which are the driving concentrations to consider for drug action. Chiou also showed that the shorter the terminal half-life, the greater the arteriovenous concentration difference during the distribution/redistribution phase. BPA’s half-life is short, and it makes sense to postulate that the peripheral venous blood BPA concentration measured following buccal absorption could systematically underestimate the arterial BPA concentration.

Finally, our article points to new alternative hypotheses for interpretation of the current epidemiological urinary data and also for better understanding blood BPA concentrations. First, the bioavailability of BPA is now theoretically up to 100% rather than about 1%, as expected following oral gavage. Second, given the typical feeding behavior of humans, the lack of uniformity of blood BPA concentrations after buccal absorption should not be ignored in terms of the PK/​PD relationship. We are now exploring the hypothesis that sublingual absorption can lead to a possible first-pass disrupting effect on pituitary secretions.
